# Dipeptide PA3264 derived from rare and endangered Squama Manis is a novel bioactive peptide for the treatment of triple-negative breast cancer

**DOI:** 10.1186/s13020-024-00979-x

**Published:** 2024-08-21

**Authors:** Xiaorong Hou, Zhaofang Bai, Yuanyuan Chen, Wei Shi, Huijie Yang, Ruisheng Li, Xiaoyan Zhan, Youping Liu, Xu Zhao, Xiaohe Xiao

**Affiliations:** 1 School of Pharmacy/School of Modern Chinese Medicine Industry, Chengdu University of Traditional Chinese Medicine, Chengdu, 611137 China; 2 Department of Hepatology, Fifth Medical Center of Chinese PLA General Hospital, Beijing, 100039 China; 3 Military Institute of Chinese Materia, Fifth Medical Center of Chinese PLA General Hospital, Beijing, 100039 China; 4 Research Institute of Department of Infectious Diseases, Fifth Medical Center of Chinese PLA General Hospital, Beijing, 100039 China

**Keywords:** Squama Manis (pangolin scale), Bioactive peptide, Traditional Chinese medicine (TCM), Anticancer, Triple-negative breast cancer (TNBC)

## Abstract

**Background:**

Squama Manis is a valuable traditional Chinese medicine with a long history of medicinal use in the treatment of breast-related diseases. However, owing to the excessive exploitation and utilization of the resources, Squama Manis has been included in the list of rare and endangered wild animals. The conservation of the resources of Squama Manis and continuing its clinical application has become an urgent problem, and the search for small-molecule substitutes for Squama Manis is an effective way to achieve this goal. Previous studies have identified PA3264 as a possible active ingredient in Squama Manis. In this study, we systematically investigated the pharmacological effects and mechanisms of PA3264 in the treatment of triple-negative breast cancer (TNBC), a representative breast-related disease.

**Methods:**

Cell viability and colony formation assays were performed after treatment with the target dipeptide PA3264 in vitro. Next, 4T1 orthotopic tumors and humanized PBMC-CDX mouse models were generated to examine the antitumor effect of PA3264 in vivo. Transcriptome sequencing and molecular docking experiments were performed to predict pathways to function. Western blotting and quantitative real-time PCR were used to validate the molecular mechanisms underlying the anticancer effects of PA3264.

**Results:**

PA3264 significantly inhibited cell viability and migration of breast cancer cells in vitro. Furthermore, PA3264 suppressed the tumor size and reduced the tumor weight in vivo. Finally, it was verified that PA3264 prevented the progression of breast cancer by inhibiting the PI3K/AKT/NF-κB pathway, causing cell cycle arrest, and promoting apoptosis.

**Conclusions:**

This study elucidated that PA3264 derived from rare and endangered Squama Manis was a novel bioactive peptide for treating triple-negative breast cancer from a scientific research perspective.

**Supplementary Information:**

The online version contains supplementary material available at 10.1186/s13020-024-00979-x.

## Introduction

Squama Manis (pangolin scale) from the scaly armor of *Manis pentadactyla Linnaeus* has a long history of medicinal use in traditional Chinese medicine. It was first recorded in the "*Ming Yi Bie Lu*" by Tao Hongjing of the Southern Dynasty and began to be known as “Chuan shanjia” in the classic works of Chinese medicine [1]. It has a salty flavor and is mildly cold. It has the function of activating blood and resolving mass, unblocking the meridian and promoting lactation, and dispersing swelling and expelling pus, et al. [2]. Squama Manis has been formulated as an ingredient of TCM prescriptions to treat patients with symptoms as amenorrhea, abdominal mass, inhibited lactation, abscess and sore, especially widely used in breast related diseases [3], including mastitis [4], breast hyperplasia [5], and breast cancer [6].

Owing to its significant food and medicinal value, Squama Manis has been subjected to destructive and indiscriminate hunting. Squama Manis is an exceptionally ecologically specialized monotypic order and family, which cannot be successfully reared and bred on a large scale commercially around the globe and can only be captured in the wild, thus leading to a drastic reduction in wild resources [7]. Squama Manis is one of the wild medicinal species protected under China's Regulations on the Protection and Management of Wild Medicinal Resources, which has been elevated from the second level of national critical protection to the first level since 2020 and has been classified as critically endangered by the International Union for Conservation of Nature (IUCN) Red List of Threatened Species [7, 8]. Therefore, it was no longer included in the 2020 edition of the Chinese Pharmacopoeia. Although the clinical efficacy of Squama Manis is exact, it has attracted considerable attention both domestically and abroad because of its endangered resources. Hence, resource protection and the search for alternatives to Squama Manis with similar efficacies are of great significance.

A great deal of work has also been carried out on the chemical composition of Squama Manis and its pharmacological effects, including anticoagulant, antithrombotic, and antitumor [1]. It has been reported that cyclic dipeptides are the main active ingredients, and L-serine-L-tyrosyclic dipeptide, D-serine-L-tyrosyclic dipeptide, L-glycine-L-tyrosyclic dipeptide, and other constituents have been successfully isolated from Squama Manis [9]. The discovery of more active peptides for treating breast-related diseases and the exploration of their mechanisms need to be followed by further studies. Previous studies have identified a peptide, PA3264, as a possible active ingredient of Squama Manis, but its pharmacological activity in breast diseases is unknown.

Triple-negative breast cancer (TNBC), a typical breast disease, is an aggressive subtype of breast cancer with recurrence and metastasis and has a poor prognosis [10]. The PI3K/AKT pathway has been reported to be a frequent signaling pathway in TNBC, making it one of the most important signaling pathways for therapeutic intervention. In addition, abnormal activation of nuclear factor-κappa B (NF-κB) induces the expression of target genes that participate in cell cycle regulation, cell proliferation, and apoptosis [11, 12]. Thus, inhibition of the PI3K/AKT pathway, as well as the downstream NF-κB pathway, contributes to antitumor effects. Here, a new linear dipeptide, PA3264, from Squama Manis, had a significant inhibitory effect on TNBC. Mechanistically, it inhibited the proliferation of breast cancer cells, caused cell cycle arrest, and promoted apoptosis by inhibiting the PI3K/AKT/NF-κB pathway. This study provides new ideas for the search for Squama Manis substitutes and the conservation of its resources. It provides a theoretical basis for a novel and effective leading compound, PA3264, for treating TNBC.

## Materials and methods

### Materials

D-Tyrosine-L-serine (PA3264) was synthesized and purified by GenScript Company (C216JHB100_5, NJ, USA). The HPLC purity was ≥ 98.0%. The peptide was soluble in ddH_2_O and stored at −20 °C. A patent application was filed for the compound (Chinese patent application for authorization No.: CN 114315958 B).

### UPLC-MS/MS analysis of PA3264 from Squama Manis

Squama Manis was obtained from Fifth Medical Center of Chinese PLA General Hospital and identified by Xiaohe Xiao professor. A Vanquish Flex UPLC system (Thermo Scientific, MA, USA) interfaced with a Q Exactive Focus mass spectrometer (Thermo Scientific) was used for UPLC-MS/MS analysis [13–15]. The following UPLC condition was used: The chromatographic column a Thermo Scientific Accucore HILIC column (2.1 × 100 mm, 2.6 μm, Part No.: 17526–102130); and an eluent of 10 mM ammonium acetate in 10% acetonitrile (A) and 10 mM ammonium acetate in acetonitrile (B) with the following gradient elution system: 25% A and 75% B in 0–6 min, then 45% B in 6–7 min, 30% B in 7–10.1 min, 75% B in 10.1–22 min at an eluent flow rate of 0.3 mL/min. The column temperature was maintained at 40 °C. The total running time was 22 min. An injection volume of 5 μL was used for each sample. The optimized parameters of MS were as follows: Spray voltage, 3.8 kV; Aux gas heater temperature, 350 °C; Sheath gas flow rate, 45 arb; Aux gas flow rate, 15 arb; Capillary temperature, 320 °C; S-lens RF, 60 V; scan mode: (1) full MS: resolution: 70, 000; automatic gain control target: 1.0e6; maximum injection time: 100 ms; scan range: 133.4–2000 m/z; (2) dd-MS2: resolution: 17500; automatic gain control target: 1.0e5; maximum injection time: 50 ms; loop count: 3; isolation window: 2.0 m/z; NCE/stepped: 20 40 60; dynamic exclusion: 8 s.

### Cell cultures

The breast cancer cell lines MDA-MB-231 and 4T1 were obtained from ATCC and maintained in high-glucose Dulbecco Modified Eagle Medium (DMEM) and RPMI 1640 medium supplemented with 100 U/mL penicillin, 100 µg/mL streptomycin (all from BOSTER, Beijing, China), and 10% fetal bovine serum (C04001-500, VivaCell, Beijing, China) in a humidified atmosphere of 5% CO_2_ at 37 °C for serial passaging. The cells were harvested during the logarithmic growth phase for subsequent experiments. The cells were frozen in a serum-free cell freezing medium (03.17004DA, EallBio, Beijing EallBio Biomedical Technology Co., Ltd, China).

### Mice

Female BALB/c mice (6–8 weeks old; 18–20 g) were purchased from SPF Biotechnology Co., Ltd. (Beijing, China) and housed in a standard pathogen-free (SPF) standard room. Six-week-old female supra-immunodeficient NCG-hIL15 mice were purchased from GemPharmatech (Jiangsu, China) and were maintained in microisolator cages under pathogen-free conditions. Laboratory animal production license: SCXY (Jing) 2019-0010, Laboratory animal production license: SCXK (Su) 2020–0004. All mice were given free access to standard laboratory chow and water ad libitum for the experiment with a 12-h light–dark cycle at a temperature of 21–25 °C. Protocols for animal experiments were approved by the guidelines for the care and use of laboratory animals. All experiments were performed under the approved guidelines of the Animal Ethics Committee of the Fifth Medical Center, Chinese PLA General Hospital (IACUC-2023-0014).

### Tumor challenge and treatment experiments

4T1 orthotopic tumors were induced via subdermal inoculation of 2 × 10^5^ 4T1 early passage cells suspended in 100 μL of PBS into the 4th mammary fat pad of BALB/c females, as described previously [16]. At 1-week post-inoculation (tumors were palpable around 15 mm^3^), the animals were randomly distributed into the control and treatment groups. PA3264 (100 mg/kg) was administered intraperitoneally (i.p.) once daily. Cisplatin (HY-17394, MedChemExpress, NJ, USA) was used as the positive control (2 mg/kg, once every 4 days) [17]. Animal body weight and tumor growth were measured three times per week. Tumor volume was calculated using the following equation: 0.5 × length × width^2^ [18]. At the end of the experimental period, the mice were sacrificed, and the remaining tumors were harvested for weighing and other experiments.

The humanized PBMC-CDX mouse model was established previously [19, 20]. MDA-MB-231 tumor cells were subcutaneously inoculated into tumor-donor mice. After the tumors grew, tumors with a volume of approximately 500–1000 mm^3^ were removed under aseptic conditions, cut into tumor masses of approximately 8 mm^3^, and inoculated subcutaneously in the right flank of experimental mice with a cannula needle. Each mouse was injected with a tumor mass. Three days after tumor inoculation, healthy adult PBMCs were inoculated into the mice by resuspension in PBS at an inoculation rate of 2 × 10^6^/mouse. Tumors were administered in groups when they grew to approximately 50–70 mm^3^ (Day10). PA3264 was intraperitoneally injected at a dose of 50 mg/kg for 21 days.

### Histology and immunohistochemistry (IHC)

Tumor samples were fixed in 4% paraformaldehyde and then dehydrated and embedded with paraffin for H&E staining. Proteins in tumor tissues were also assessed by immunohistochemistry assay. Tissue sections (4 μm) were deparaffinized, rehydrated, and antigen-repaired. After blocking, anti-Ki67 (Abcam, ab16667, 1:100) and Caspase 3 (Bioss, bs-0081R, 1:100) were incubated overnight at 4 °C. The corresponding secondary antibody was incubated for 1 h. Finally, sections were stained with 3,3'-diaminobenzidine (DAB, BOSTER, AR1022), counter-stained with hematoxylin, dehydrated with gradient ethanol, cleared with xylene, sealed with neutral gum, observed, and photographed under an Eclipse microscope (Nikon Corporation). The proportion of positively stained area was analysed using Image J by calculating the positive areas in the total cells [21, 22].

### Transcriptome sequencing (RNA-seq) analysis

Total RNA was extracted from the samples and enriched with oligo (dT)-attached magnetic beads. The synthesized and purified double-stranded cDNA was subjected to end repair and 3'-adenylation. Sequencing adapters were connected to the ends of these 3′ adenylated cDNA fragments, and the selected fragments were amplified by PCR and qualified using an Agilent 2100 Bioanalyzer (Agilent Technologies, Santa Clara, CA, USA). Libraries were constructed using the VAHTS Universal V6 RNA-seq Library Prep Kit according to the manufacturer’s instructions after the quality inspection was completed. Sequencing was performed using Illumina HiSeqTM 2500. Differential expression analysis was performed using the DESeq2. A Q value < 0.05 and foldchange > 2 or foldchange < 0.5 was set as the threshold for significantly differentially expressed genes (DEGs). Transcriptome sequencing and analysis were performed by OE Biotech Co. Ltd. (Shanghai, China) using standard procedures.

### Molecular docking

Molecular docking was performed using Schrödinger Suite Release 2018-1 [23, 24]. PI3Kγ, AKT1, and NF-κB p65 proteins were used as receptors, and PA3264 was used as a ligand. Three-dimensional crystal structures of PI3Kγ (PDB ID: 4HVB), AKT1 (PDB ID: 4EKL), and NF-κB p65 (PDB ID: 1NFI) were retrieved from the RCBS PDB database. This structure was optimized before docking using Protein Preparation Wizard in Maestro 11.5. The docking of the receptor (target) preparation involves the deletion of water molecules from target proteins, removal of protein polymorphisms, complementation of non-complete amino acid residues, and hydrogenation of proteins, followed by lattice-box construction of each target protein. LigPrep was used to convert structures from 2-dimensional to 3-dimensional, correction improper bond distances and bond orders, generating ionization states, and minimizing energy. The processed protein and ligand structures were subjected to docking analysis. Finally, docking scores were calculated to evaluate the binding activity between the ligand and three targets. The display diagram of the molecular docking results was modified using PyMOL.

### Cell viability assay

To assess the effects of PA3264 on cell viability, 1.5 × 10^5^ MDA-MB-231 and 9.0 × 10^4^ 4T1 cells per well in 96 well plates were incubated for 12 h. The medium was then replaced with different PA3264 concentrations (0, 0.078, 0.156, 0.313, 0.625, 1.25, 2.5, 5, 10, and 20 mg/mL) for the indicated times. Cell viability was measured using the CCK-8 assay kit (G4103-5ML, Servicebio, Wuhan, China) according to the manufacturer’s instructions. The optical density (OD) values used as the index of cell viability were measured at 450 nm using a microplate reader, and the IC_50_ values were calculated as described previously [21]. Lactate dehydrogenase (LDH) is a stable cytoplasmic enzyme in all cells. LDH is rapidly released into the cell culture supernatant when the plasma membrane is damaged, a key feature of cells undergoing apoptosis, necrosis, and other forms of cellular damage [25]. The LDH release assay was performed using the LDH Cytotoxicity Assay kit (G1610-100 T, Servicebio, Wuhan, China), according to the manufacturer's protocols.

### Colony formation assay

To assess clonogenic ability, single cell suspensions containing 2 × 10^3^ MDA-MB-231 and 4T1 cells were seeded in 12-well plates and allowed to adhere for 24 h, and treated with PA3264 for 5 days. The cells were fixed in 4% paraformaldehyde and stained with crystal violet. Finally, the colonies were quantified using the ImageJ software.

### Wound healing assay

A total of 2 × 10^5^ cells were seeded in the wells of a 12-well plate for 24 h. The cell layer was scratched using a sterile pipette tip (200 µL). Subsequently, cells were cultured with the indicated concentrations of PA3264. Representative images of the wounds were taken at 0 and 24 h using an Eclipse microscope (Nikon Corporation, Tokyo, Japan). Each experiment was conducted in triplicate.

### Quantitative real-time PCR (qRT-PCR)

Total RNA was extracted using TRIzol reagent (Alcatel, Beijing, China), and the isolated RNA was reverse-transcribed into cDNA using StarScript III All-in-one RT Mix with gDNA Remover (GenStar, Beijing, China). Primer sequences used are listed in Table 1. Quantitative real-time PCR was performed on a QuantStudio 6 (96-well format, Thermo Fisher, USA) using SYBR Green qPCR Master Mix (Low ROX) (GenStar, Beijing, China). The PCR conditions were as follows: 95 °C for 5 min, followed by 40 cycles of 95 °C for 10 s, and 60 °C for 60 s. The expression levels of these genes were normalized to that of β-actin. The data were analyzed and displayed using the ΔΔCt or 2^−ΔΔCt^ method.

### Western blotting

Cell lysates were collected using RIPA buffer [50 mM Tris–HCl, 150 mM NaCl, 1% sodium deoxycholate, and 1% Triton-100, pH 7.45]. Protein denaturation was performed before loading at 100 °C for 15 min. Proteins were separated using 10% SDS-PAGE and transferred to PVDF membranes (Millipore Corp., Bedford, MA, USA). Membranes were then blocked with 5% skim milk for 1 h and exposed to the primary antibodies with an appropriate dilution at 4 °C overnight: p-PI3K (Abcam, ab278545,1: 1500), PI3K (ZENBIO, R22768, 1: 2000), p-AKT (Cell Signaling Technology, 4060 T, 1: 2000), AKT1 (HUABIO, ET1609-47, 1: 1500), p-p65 (Abcam, ab76302, 1: 1500), p65 (Cell Signaling Technology, 8242S, 1: 1000), Cyclin D1 (Abcam, ab134175, 1: 5000). HSP90 (ZENBIO, 251211, 1:2000) was used as the internal control. Membranes were subsequently probed with anti-mouse or anti-rabbit IgG antibodies conjugated to horseradish peroxidase (Transduction Laboratories, Lexington, KY, USA) and visualized using enhanced chemiluminescence (ECL, E422-02, Vazyme, Nanjing, China).

### Quantification and statistical analysis

Two-tailed unpaired Student’s t-test was used to compare two groups. Multiple groups were tested using a one-way analysis of variance (ANOVA). Two-factor ANOVA was used for tumor growth analysis. All data are presented as mean ± SEM. Data were processed using GraphPad Prism version 9 (GraphPad Software). In all cases, *p* values of 0.05 and below were considered to be statistically significant: *p* < 0.05 (*), *p* < 0.01 (**), and *p* < 0.001 (***) were considered statistically significant. Detailed information regarding the number of replicates can be found in the figure legend.

## Results

### UPLC-MS/MS analysis of PA3264 from Squama Manis

**Fig. 1:**
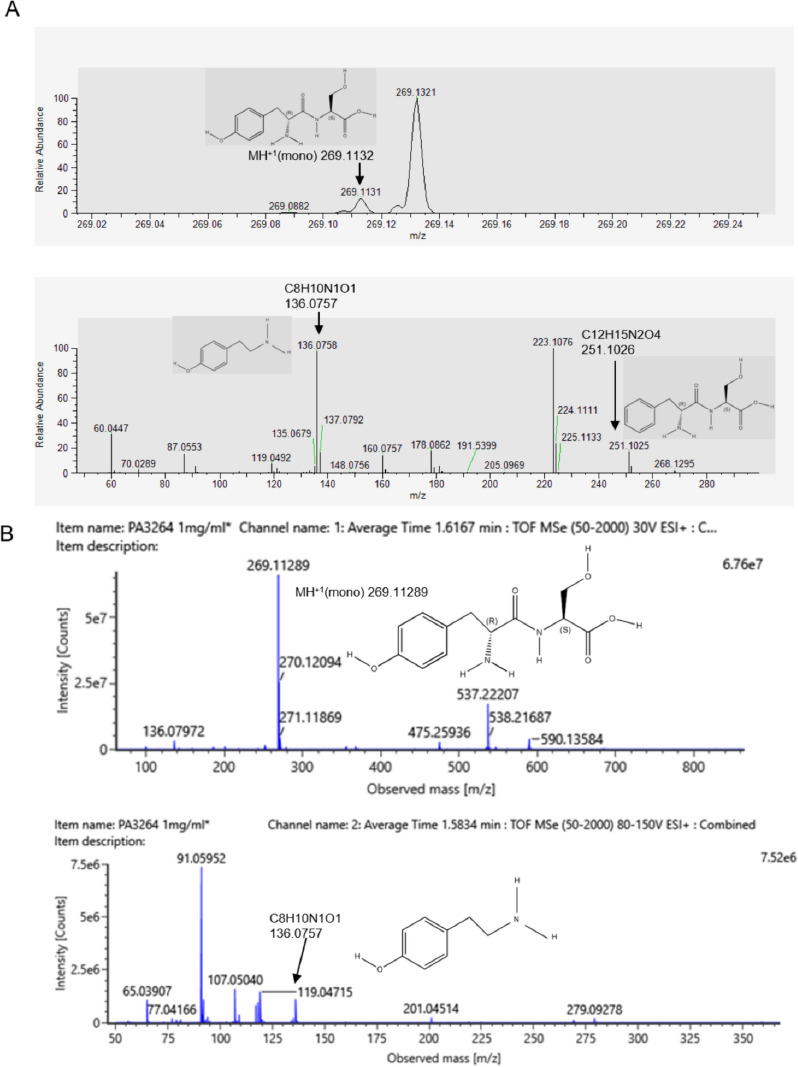
UPLC-MS/MS analysis of PA3264 from Squama Manis. **A** The upper panel shows the extracted ion chromatogram of PA3264. The mass spectra of PA3264 are shown in the lower panel. **B** MS and MS/MS spectra of synthetic dipeptide PA3264

The extracted ion chromatogram of PA3264 from Squama Manis showed that its molecular formula of PA3264 was C12H16N2O5, with a theoretically calculated [M + H] + mass charge ratio (m/z) of 269.1132. The lower image characterized the possible structures of the two fragment ions in the mass chromatogram of PA3264 (Fig. 1A). The mass spectral verification of the synthetic dipeptide PA3264 was shown in Fig. 1B. The theoretically calculated [M + H] + mass-to-charge ratio (m/z) was 269.11289. One of the fragment ions matched the one shown in Fig. 1A. The structure of PA3264 was roughly deduced from the molecular ion peaks and fragment ion results of PA3264 in Squama Manis and synthetic peptide samples. Next, we investigated the pharmacological activity and mechanism of action of PA3264 in breast-related diseases represented by triple-negative breast cancer.

### PA3264 restrained the viability and clonogenicity in murine and human-derived breast cancer cells

**Fig. 2:**
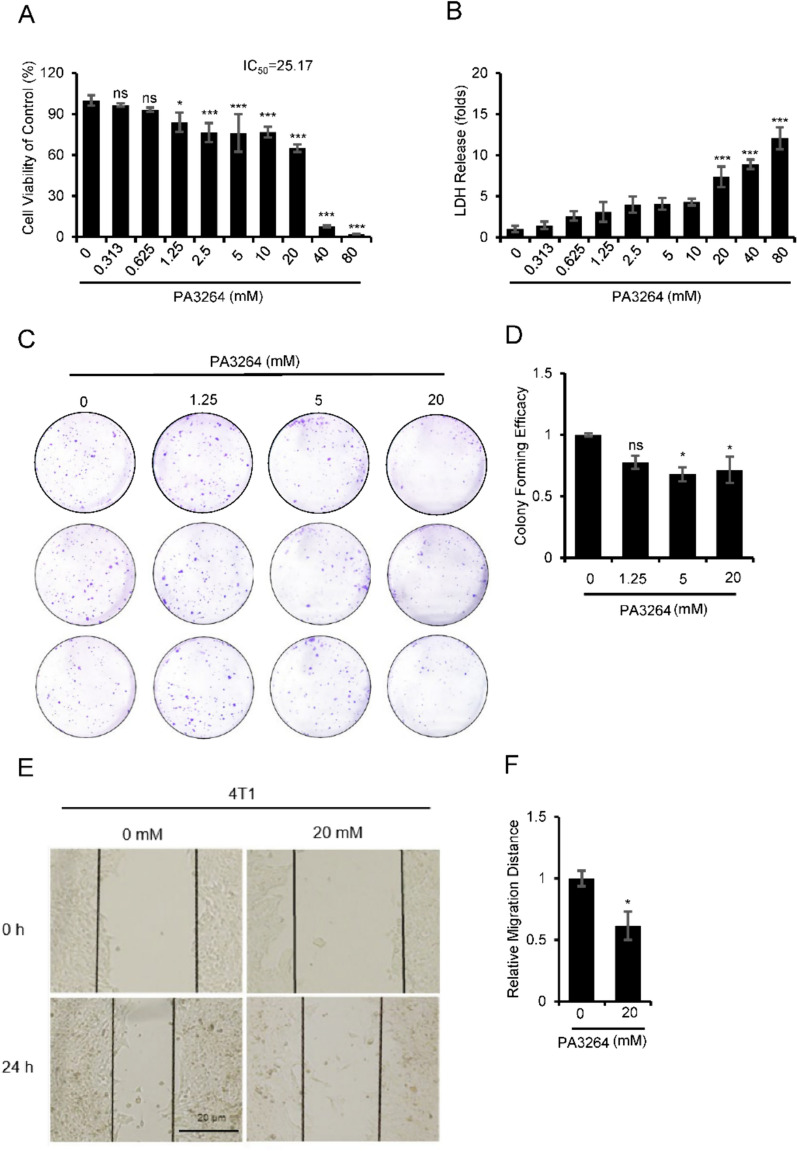
PA3264 inhibited the proliferation and migration of 4T1 cells. **A** Inhibition of 4T1 cell proliferation was assessed by the CCK-8 assay after 24 h of treatment with different concentrations of PA3264. **B** LDH was released from 4T1 cells after 24 h of treatment with different concentrations of PA3264. **C** Colony formation after treated with PA3264 at different concentrations (0, 1.25, 5, and 20 mM) using a plate cloning assay. **D** Quantitative histogram of the clone formation. **E**–**F** Representative images and results of wound healing assays with 4T1 cells treated with PA3264 (0, 20 mM) for 24 h. The figure shows the mean ± SEM of the experimental data for each group. *p < 0.05, **p < 0.01, ***p < 0.001 (versus the control group), n = 3

**Fig. 3:**
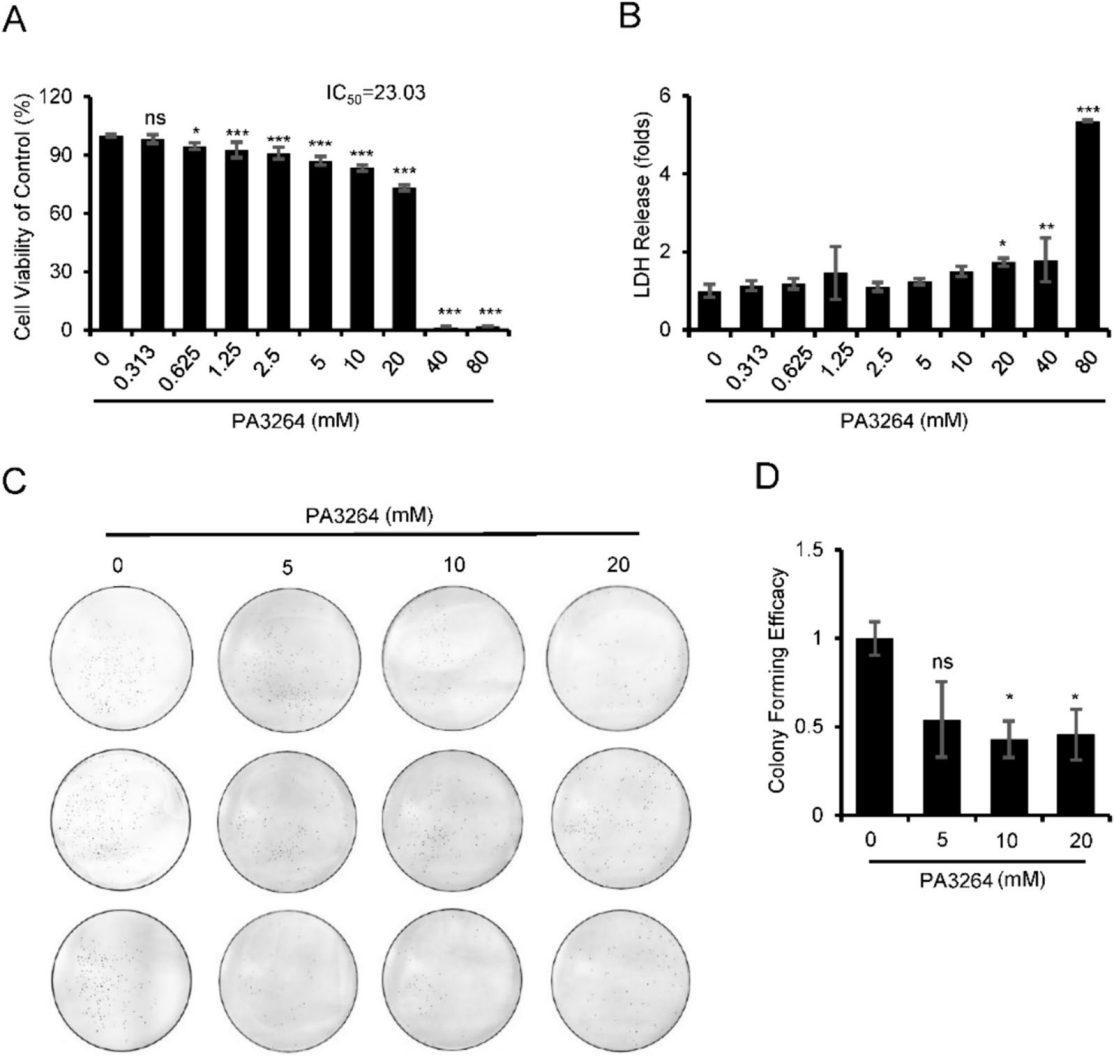
PA3264 significantly suppressed the viability of MDA-MB-231 cells. **A** MDA-MB-231 cells were treated with serial concentrations of PA3264, and the effects of PA3264 on cell proliferation were measured using the Cell Counting Kit-8 examinations at 24 h. **B** LDH was released from MDA-MB-231 cells after 24 h of treatment with different concentrations of PA3264. **C**–**D** Images and results of colony formation assay with MDA-MB-231 cells treated with PA3264. Data were presented as mean ± SEM, n = 3, *p < 0.05, **p < 0.01, ***p < 0.001 vs. the control group

To explore the effect of PA3264 on the proliferation of murine and human breast cancer cells, CCK-8 and LDH release assays were used to determine the viability of 4T1 and MDA-MB-231 cells after treatment with various concentrations of PA3264 for 24 h. The results showed that the proliferation of 4T1 and MDA-MB-231 cells treated with PA3264 was significantly lower than that of the control group, in a dose-dependent manner (Figs. 2A and 3A). The LDH release assay showed that PA3264 treatment accelerated plasma membrane damage in breast cancer cells in a dose-dependent manner (Figs. 2B and 3B). In addition, colony formation assays showed a decrease in the number of colonies formed as the intervention concentration of PA3264 increased (Figs. 2C, D and 3C, D). These results indicated that PA3264 curbed the cell viability and colony formation of murine and human-derived breast cancer cells in a dose-dependent manner.

### PA3264 constrained the migration of 4T1 cells

To evaluate the effect of PA3264 on 4T1 cell migration, wound healing assays were performed. After 4T1 cells were incubated with 20 mM PA3264 for 24 h, the migration distance was significantly reduced by 39% compared with the control group (Fig. 2E, F, *p* < 0.05). The data demonstrated that PA3264 had a remarkable prevention on wound healing as an inhibitor of 4T1 cell migration.

### PA3264 inhibited tumor growth in vivo

**Fig. 4:**
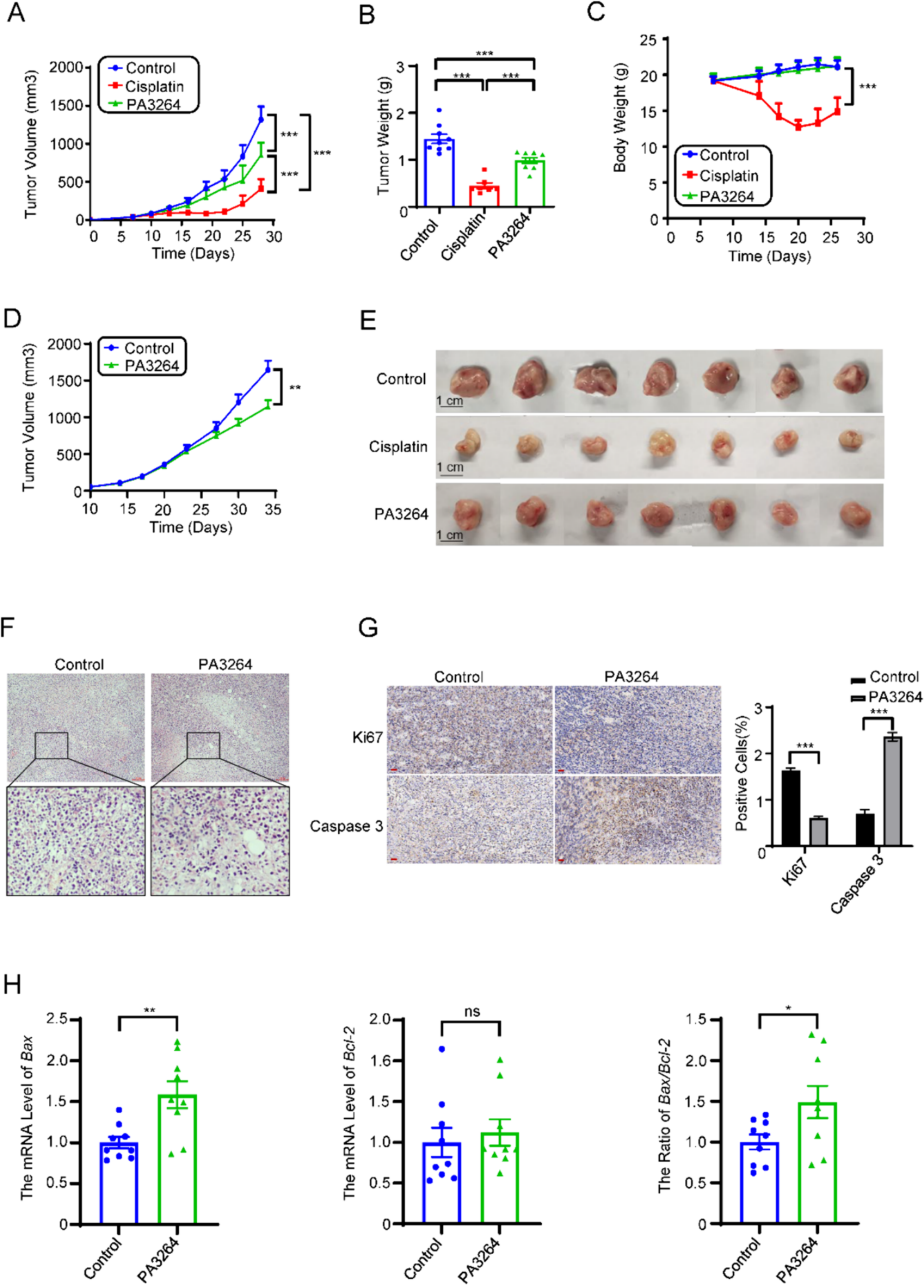
PA3264 inhibited tumor growth in vivo by inhibiting proliferation and inducing apoptosis. **A** PA3264 suppressed tumor growth in mice bearing 4T1 orthotopic tumors. **B** PA3264 decreased the tumor weight in mice bearing 4T1 orthotopic tumors. **C** The body weights of mice were checked every three days. Data were presented as the mean ± SEM (n = 7–9). **D** Growth curve of tumor volumes in humanized PBMC-CDX mouse model. Data were presented as mean ± SEM, n = 6. **E** Tumors of 4T1 cells treated with PA3264 or saline for 21 days were photographed. **F** Representative H&E-stained histological images of tumor sections. Scale bar: 10 nm, n = 5. **G** Representative histological images of tumor sections stained with Ki67 and Caspase 3 and quantitative analysis of their expression. Scale bar: 20 μm, n = 5. **H** *Bax* and *Bcl-2* mRNA expression in tumor tissues was analyzed using quantitative real-time PCR. The ratio of *Bax/Bcl-2* expression was also shown. Data were presented as mean ± SEM, n = 9. *p < 0.05, **p < 0.01, ***p < 0.001 vs. the control group

To examine the antitumor effects of PA3264 in vivo, 4T1 orthotopic tumors were constructed and treated. As shown in Fig. 4A, B, tumor volume and weight in the model group increased more dramatically. At the same time, cisplatin and PA3264 treatments significantly inhibited the increase in tumor volume and weight as early as 4 weeks after injection. At the end of this period, the tumor volume and weight in PA3264-treated mice were reduced by 41% and 32%, respectively, compared to those in control mice (*p* < 0.01). Cisplatin used as a positive control had a significant depression effect on tumor size and weight. Strikingly, the treated groups had a smaller burden than the control group (Fig. 4E). In addition, a humanized PBMC-CDX mouse model was used to validate the antitumor activity of the PA3264. Tumor volume was also reduced in mice treated with PA3264 for 24 consecutive days compared with the control group (Fig. 4D, p < 0.01).

Body weight is one of the most important indicators for evaluating the safety of anticancer drugs. Throughout the experimental period, the body weight of mice treated with PA3264 did not change significantly compared with that of the control group (Fig. 4C). However, the body weights of cisplatin-treated mice decreased significantly 3 weeks after injection compared with the control group (Fig. 4C). Additionally, the effect of PA3264 on liver and renal function indicators in 4T1 tumor-bearing mice was investigated by measuring serum related biochemical parameters (ALT, AST, BUN, and CRE). Compared with the normal group, the serum BUN levels in the model group were significantly elevated (*p* < 0.01), indicating that the renal function of the tumor-bearing mice was damaged to a certain extent (Suppl. Figure 1C). After cisplatin treatment in the tumor-bearing mice, the levels of serum ALT, BUN, and CRE in the cisplatin group were significantly higher than those in the control group (*p* < 0.05, Suppl. Figure 1A, 1C, and 1D), while the levels of AST were also increased (*p* > 0.05, Suppl. Figure 1B), indicating that the liver and renal function of the tumor-bearing mice were damaged to a certain extent by cisplatin administration. After treatment with PA3264 in the tumor-loaded mice, the levels of ALT, AST, BUN, and CRE were lower than those in the model group, but the difference was not statistically significant (*p* > 0.05, Suppl. Figure 1A–D). Compared with the cisplatin group, the levels of ALT, BUN, and CRE in the PA3264-treated group were significantly decreased (*p* < 0.01, Suppl. Figure 1A, 1C, and 1D), while the levels of AST were also reduced. Still, the difference was not statistically significant (*p* > 0.05, Suppl. Figure 1B). This suggested that PA3264 did not significantly damage the liver and kidney function of the tumor-loaded mice. In summary, treatment with PA3264 and cisplatin was largely effective in regressing tumors and PA3264 was safer to administer in vivo than cisplatin.

The histology of tumor sections showed that tumor cells, which had apparent nucleoli and large nuclei, were arranged tightly in the control group. In contrast, there was a loose arrangement in the PA3264 treated group (Fig. 4F). In addition, immunohistochemical staining of tumor tissues with Ki67 and Caspase 3 was performed. PA3264 administration decreased the expression of Ki67 and increased the expression of Caspase 3 in the tissues compared with the control group (Fig. 4G). Quantitative real-time PCR analysis of tumor tissues showed that the expression of *Bax* and the *Bax*/*Bcl-2* ratio was upregulated after PA3264 treatment (Fig. 4H). Taken together, these in vivo data suggest that PA3264 inhibited the proliferation and promoted the apoptosis of triple-negative breast cancer.

### Identification of DEGs and their pathways in PA3264-mediated tumor suppression by RNA-seq analysis

**Fig. 5:**
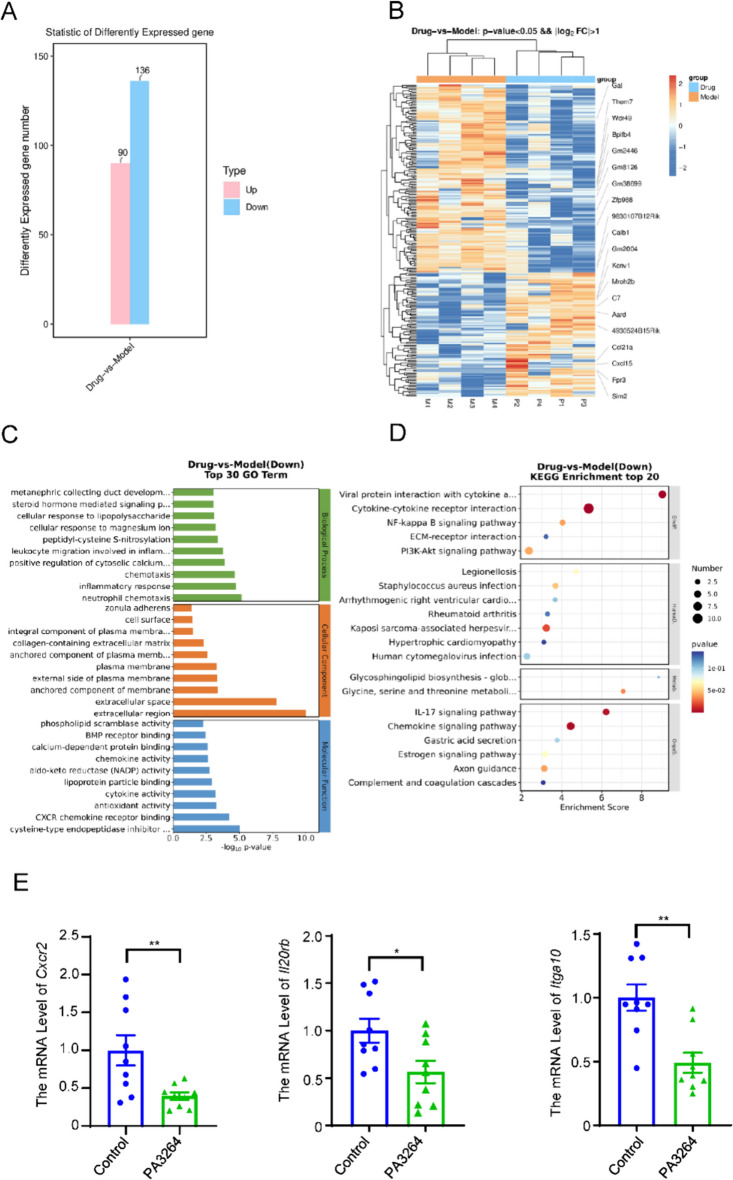
RNA-seq analysis of PA3264 in 4T1 orthotopic tumors. **A** The bar chart showed the number of upgraded or downgraded DEGs in tumors between the control and PA3264-treated groups. **B** Clustering heatmap analysis of DEGs in different groups using RNA-seq analysis. **C** GO enrichment analysis of the candidate targets of PA3264 in the treatment. The top 10 GO functional categories were selected. **D** KEGG pathway enrichment of the candidate targets of PA3264 in treating TNBC. The size of the circles represents the number of genes, and the color represents the *p-value*. **E** qRT-PCR verification of DEGs related to the PI3K/AKT signaling pathway. Data were presented as mean ± SEM, n = 9, *p < 0.05, **p < 0.01, ***p < 0.001 vs. the control group

To explore the genes and pathways involved in inhibiting 4T1-induced triple-negative breast cancer by PA3264, RNA from the four replicate samples from the control and PA3264-treated groups were sequenced. Cutoff values were set at |log2 fold change (FC)|> 1 and *p* < 0.05. A clustering heatmap and volcanic distribution of the DEGs were obtained. A total of 226 differential expression genes (DEGs) were identified. Overall, 90 upregulated and 136 downregulated DEGs were identified in the control and PA3264-treated groups, respectively (Fig. 5A). Hierarchical clustering analysis of the DEGs between the control and PA3264-treated groups was presented as a heat map, indicating significant differences in the expression patterns of the DEGs between the two groups (Fig. 5B). The DEGs were subjected to GO enrichment analysis to characterize their functions. Results showed that these DEGs were significantly enriched in GO functions associated with extracellular regions, extracellular space, neutrophil chemotaxis, and cysteine-type endopeptidase inhibitor activity (Fig. 5C). Moreover, KEGG pathway analysis showed that the DEGs were mainly associated with cytokine-cytokine receptor interaction, NF-kappa B signaling pathway, ECM-receptor interaction, PI3K-AKT signaling pathway, IL-17 signaling pathway, and chemokine signaling pathway in control vs. PA3264-treated groups (Fig. 5D). Furthermore, some DEGs related to the PI3K/AKT signaling pathway with significant fold differences in expression were verified by qRT-PCR. The results were consistent with the RNA-seq results (Fig. 5E).

**Table 1:** Primer sequences of target genes

Gene	Forward primer	Reverse primer
h *Cyclin D1*	*GCTGCGAAGTGGAAACCATC*	*CCTCCTTCTGCACACATTTGAA*
h *Bcl-2*	*AGTTCGGTGGGGTCATGTGT*	*CCAGGAGAAATCAAACAGAGGC*
h *β-actin*	*CATGTACGTTGCTATCCAGGC*	*CTCCTTAATGTCACGCACGAT*
m *Cyclin D1*	*TGACTGCCGAGAAGTTGTGC*	*CTCATCCGCCTCTGGCATT*
m *Bcl-2*	*GCTACCGTCGTGACTTCGC*	*CCCCACCGAACTCAAAGAAGG*
m *Bax*	*AGACAGGGGCCTTTTTGCTAC*	*AATTCGCCGGAGACACTCG*
m *Itga10*	*GACTGTGGCCCTGACAATGA*	*AAATGGGGACTTCCTGGAGC*
m *Cxcr2*	*TCGTAGAACTACTGCAGGATTAAG*	*GGGACAGCATCTGGCAGAATA*
m *Il20rb*	*TGTGGAGTATCAGGGGGAGT*	*TGGTCGGTGAGCATTGACTG*
m *β-actin*	*GGCTGTATTCCCCTCCATCG*	*CCAGTTGGTAACAATGCCATGT*

### Molecular docking

**Fig. 6:**
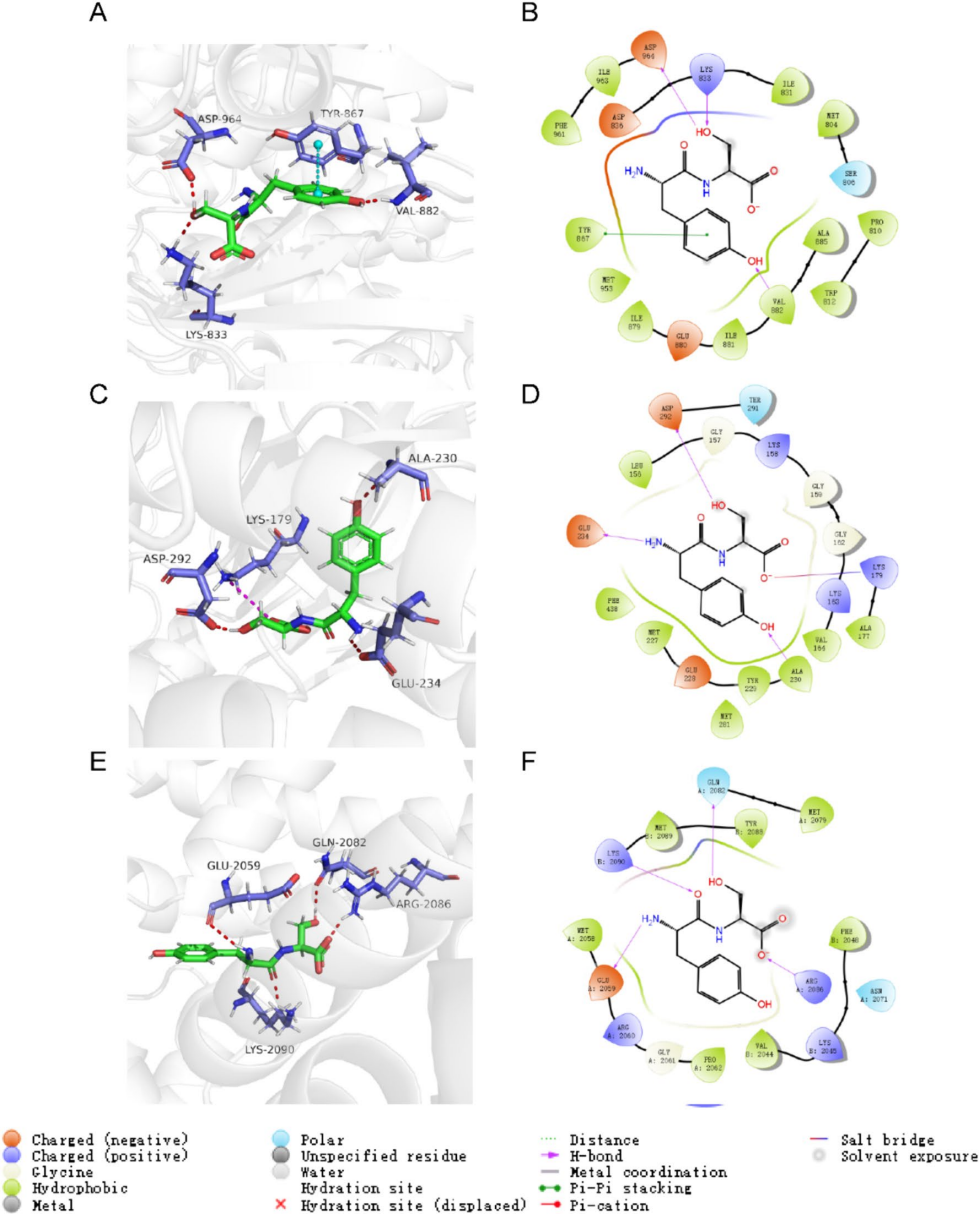
PA3264 docked in the binding sites of macromolecular targets. i) PA3264 docked to the binding site of PI3K (PDB ID: 4HVB). **A** 3D picture depicting the binding mode. **B** 2D interaction diagram showing ligand-receptor interactions; ii) PA3264 docked at the binding site of AKT (PDB ID: 4EKL) **C** 3D binding pose, **D** 2D interaction diagram; iii) PA3264 docked at the binding site of NF-κB p65 (PDB ID: 1NFI) **E** 3D binding pose, **F** 2D interaction diagram

Aberrant activation of the PI3K/AKT/NF-κB pathway is commonly observed in breast cancer, resulting in the increased proliferation and invasive potential of cancer cells. To predict the binding affinity of PA3264 for related proteins in the PI3K/AKT/NF-κB pathway, molecular docking studies were performed. PA3264 and the surrounding amino acid residues of macromolecular targets helped stabilize the complex structure, thus enhancing binding affinity. Figure 6A, B depict the interaction of PA3264 with the binding site residues of PI3Kγ. The phenyl ring of Tyr867 interacted with PA3264 via face-to-face pi-pi stacking interaction with the phenyl ring of PA3264. Additionally, the hydroxy group of PA3264 formed 3H-bonds with residues Asp967, Lys833, and Val882. Next, the molecular docking of PA3264 with AKT1 (Fig. 6C, D) and NF-κB p65 (Fig. 6E, F) was carried out. Furthermore, the molecular docking results revealed that PA3264 had the highest binding affinity with the inhibitory targets of PI3K, with a docking score of −6.987, as shown in Table 2.

**Table 2:** Docking analysis results for the compounds

Macromolecular targets	PDB ID	Docking scores
PI3Kγ	4HVB	−6.987
AKT1	4EKL	−5.466
NF-κB p65	1NFI	−4.141

### PA3264 caused cell cycle arrest and promoted apoptosis by regulating the PI3K/AKT/NF-κB signaling axis

**Fig. 7:**
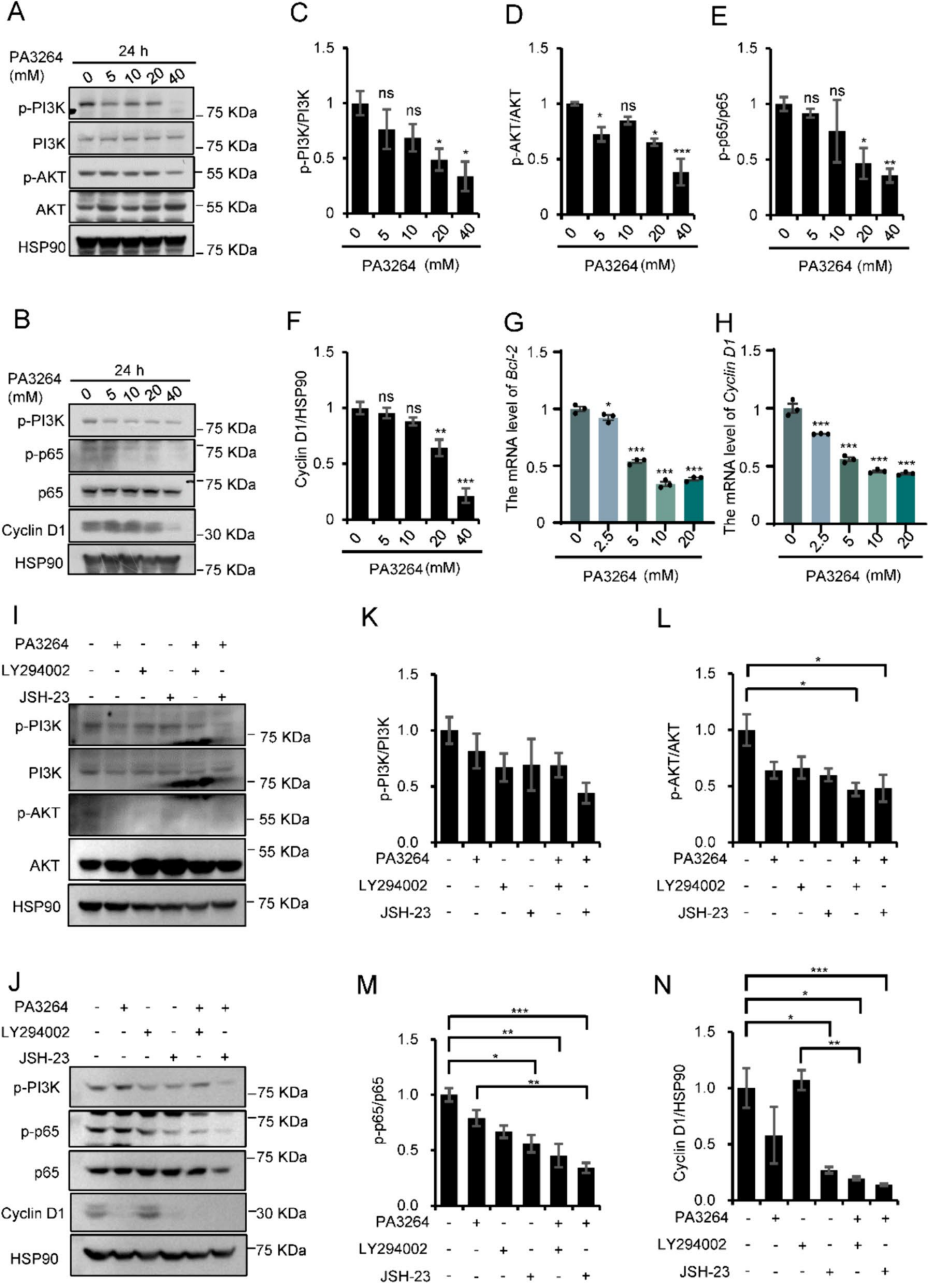
PA3264 caused cell cycle arrest and induced apoptosis in 4T1 cells via the PI3K/AKT/NF-κB signaling pathway. **A**–**B** Dose–response effects of PA3264 on signaling pathways in 4T1 cells treated with the indicated concentrations for 24 h. The levels of p-PI3K, PI3K, p-AKT, AKT, p65, p-p65, and Cyclin D1 in PA3264-treated 4T1 cells were examined using western blotting. **C**–**E** The relative intensities of all phosphorylated proteins were calculated after normalization to the total proteins. **F** Relative intensities of Cyclin D1 were calculated after normalization to HSP90 expression. **G**–**H** The expression of *Cyclin D1* and *Bcl-2* mRNA in 4T1 cells was verified by qRT-PCR. **I**–**J** 4T1 cells were treated with 20 mM PA3264, 0.1 μM LY294002, and 10 μM JSH-23 for 24 h. The expression levels of PI3K, AKT1, p65, their phosphorylated forms, and Cyclin D1 were assessed in cell lysates by western blotting analysis. **K**–**M** Relative intensities of all phosphorylated proteins were calculated after normalization to total protein. **N** The relative intensity of Cyclin D1 was calculated after normalization with HSP90 expression. Data were presented as the mean ± SEM (n = 3). *p < 0.05, **p < 0.01, and ***p < 0.001, compared with levels in the untreated group or the group administered alone

To investigate the anticancer effects of PA3264 and verify the PI3K/AKT/NF-κB pathway-dependent mechanism, we measured the expression levels of related proteins using western blotting. As shown in Fig. 7A–F, following intervention with different concentrations of PA3264 for 24 h on the 4T1 cells, the protein expression of p-PI3K, p-AKT, p-p65, and Cyclin D1 was reduced in a dose-dependent manner. And the expression levels of p-PI3K, p-AKT, p-p65, and Cyclin D1 proteins were significantly lower in the 20 mM and 40 mM groups than in the control group (*p* < 0.01 or 0.05). Consistent with the WB analysis, the mRNA expression levels of *Bcl-2* and *Cyclin D1* also gradually declined in PA3264-treated 4T1 cells compared to those in the control group (*p* < 0.01 or 0.05, Fig. 7G, H), as expected. To further validate these results, we performed the same experiments using MDA-MB-231 cells. The expression levels of p-PI3K, p-AKT, p-p65, and Cyclin D1 proteins were significantly lower in the 10 mM and 20 mM groups than in the control group (*p* < 0.01 or 0.05, Suppl. Figure 2A-F). Moreover, PA3264 treatment significantly downregulated *Bcl-2* and *Cyclin D1* expression in the 10 mM and 20 mM groups compared to that in the control group (*p* < 0.01 or 0.05), as evidenced by the mRNA expression level (Suppl. Figure 2G-H). Overall, these results indicated that PA3264 suppressed the PI3K/AKT/NF-κB signaling pathway to regulate the cell cycle and apoptosis in breast cancer cells, further validating the accuracy of RNA-seq analysis and molecular docking results.

To further validate the mechanism of PA3264 in the treatment of TNBC, PI3K and NF-κB pathway inhibitors were used in western blotting experiments. LY294002 and JSH-23 have been reported to be PI3K and NF-κB pathway inhibitors, respectively [26–29]. We investigated the inhibitory effects of PA3264 in combination with LY294002 on PI3K/AKT signaling and JSH-23 on NF-κB signaling in breast cancer cells (MDA-MB-231 and 4T1). LY294002 and PA3264 reduced p-PI3K, p-AKT, and p-p65 protein expression in comparison with the control group (Fig. 7I–M and Suppl. Figure 2I-M). Compared with the LY294002 group, the expression of p-PI3K, p-AKT and p-p65 proteins decreased with no significant difference, when PA3264 was co-administered with LY294002. Still, Cyclin D1 protein expression had a significant decreasing trend (*p* < 0.01, Fig. 7N and Suppl. Figure 2N), which indicated that PA3264 exerted a part of its anti-TNBC effect by inhibiting the PI3K/AKT signaling pathway. In addition, JSH-23 and PA3264 inhibited the expression of p-p65 and Cyclin D1 proteins in comparison with the control group. The combination of PA3264 and JSH-23 reduced the expression of p-p65 and Cyclin D1 proteins without significant difference compared with the JSH-23 group, suggesting that PA3264 played a part of its anti-TNBC effect by suppressing the NF-κB signaling pathway. Therefore, in this study, we found that PA3264 exerted a certain anti-TNBC effect by inhibiting the PI3K/AKT/NF-κB signaling pathway.

## Discussion

Squama Manis has a long history of medicinal application in China to invigorate blood circulation and remove blood stasis. Clinically, Squama Manis is used to treat a wide range of diseases with significant results, not only for breast-related diseases but also for other gynecological disorders, including fibroids [31] and infertility [32]. Although the effect of Squama Manis has been demonstrated through the accumulation of real-life experiences, the role of individual biochemically active ingredients has not been extensively investigated. Further research should be conducted to develop active compounds as alternatives to Squama Manis to protect this endangered species.

Breast cancer is one of the most common breast-related diseases with a high incidence and mortality rate. TNBC, the most aggressive subtype, quickly metastasizes, and exhibits strong drug resistance. Chemotherapeutic agents are widely used to treat patients with advanced BC. However, approximately 50% of the patients rapidly develop acquired resistance [30]. Therefore, it is still important to discover new TNBC treatments, and providing more effective treatments with fewer side effects is the primary and challenging research direction. Traditional Chinese medicine (TCM) plays an anticancer role by regulating the immune system, anti-tumor angiogenesis, tumor cell apoptosis, autophagy dysfunction, and other mechanisms. An increasing body of literature reports traditional Chinese medicine and its active constituents possess significant anticancer activity, such as Icariside II [31] and curcumin [32, 33]. Therefore, exploring new anticancer agents from TCM and their mechanisms in treating TNBC has become a research hotspot in recent years.

It is widely believed that restraining the PI3K/AKT/NF-κB pathway is a crucial target for treatment. The omnipresent PI3K/AKT pathway is involved in apoptosis, proliferation, and differentiation by regulating a wide range of target proteins, including NF-κB [34]. The phosphorylation of p65 leads to NF-κB translocation into the nucleus and binding to specific sequences in the promoter regions of target genes encoding mediators such as Cyclin D1, Bcl-2 [35, 36]. By promoting Cyclin D1 and Bcl-2 expression, the NF-κB pathway drives cell cycle progression and promotes the proliferation of cancer cells.

Previous studies have found a peptide, PA3264, derived from Squama Manis, but its pharmacological activity in breast diseases is unknown. In this study, we found that PA3264 significantly inhibited the viability of BC cells (MDA-MB-231 and 4T1) in a dose-dependent manner. In addition, mechanistic studies have suggested that PA3264 downregulated the phosphorylation of PI3K, AKT and p65, and decreased the expression of Cyclin D1 and Bcl-2, thereby inhibiting cell proliferation and inducing apoptosis. Consistent with the results of the in vitro experiment, PA3264 significantly inhibited tumor development and induced apoptosis in vivo. Treatment with PA3264 did not substantially affect the average body weight, the liver and kidney function of mice. Taken together, these results suggest that PA3264 is a potentially safe and effective anticancer treatment for TNBC.

There are two main limitations with regard to this research. The present study yielded that PA3264 inhibited cell migration only at 4T1. However, the in vivo effects need to be further investigated. Transcriptome sequencing results enriched multiple signaling pathways that may inhibit TNBC, but we focused only on the PI3K/AKT and NF-κB pathways. The current results suggest that PA3264 inhibited the activation of the PI3K/AKT/NF-κB pathway by suppressing PI3K phosphorylation. To further support this, PI3K and NF-κB pathway inhibitors were used in western blotting experiments. Therefore, in this study, we found that PA3264 exerted a certain anti-TNBC effect by inhibiting the PI3K/AKT/NF-κB signaling pathway, but other mechanisms of action also exist. More studies are needed to address these limitations in the future to characterize its therapeutic functions and elucidate other molecular mechanisms that exert its biological effects in TNBC.

## Conclusion

In this study, we preliminarily clarified the anti-tumorigenic activity and mechanisms of action of PA3264 in TNBC treatment. Our findings suggested that PA3264 inhibited tumor growth by modulating the PI3K/AKT/NF-κB signaling axis, suppressing proliferation and promoting apoptosis. Altogether, this study indicates that PA3264 as a potential leading compound has a therapeutic benefit against TNBC and promises to be a substitute for rare and endangered Squama Manis in the treatment of TNBC.

### Supplementary Information

Supplementary material 1: Fig. 1 Effects on liver and renal function indicators in various groups of mice. **A** Alanine aminotransferase (ALT), **B** aspartate aminotransferase (AST), **C** blood urea nitrogen (BUN), and **D** creatinine (CRE) levels in the serum were measured. The figure shows the mean ± SEM of the experimental data for each group. Compared with the normal and control groups, *p < 0.05, **p < 0.01, ***p < 0.001. Compared with the cisplatin group, ^#^*p *< 0.05, ^##^*p *< 0.01, ^###^*p *< 0.001, n = 7-9.

Supplementary material 2: Fig. 2 Effect of PA3264 with pathway inhibitors on PI3K/AKT/NF-κB signaling in MDA-MB-231 cells. **A-B** MDA-MB-231 cells were treated with the indicated concentrations of PA3264 for 24 h, and the expression levels of PI3K, AKT1, p65, Cyclin D1, and all phosphorylated forms in total cell lysates were evaluated by western blot analysis. **C-E** The relative intensities of all phosphorylated proteins were calculated after normalization to the total proteins. **F** Relative intensities of Cyclin D1 were calculated after normalization to HSP90 expression. **G-H** Analysis of Cyclin D1 and Bcl-2 expression in MDA-MB-231 cells treated with PA3264 at different concentrationsusing qRT-PCR. **I-J** MDA-MB-231 cells were treated with 20 mM PA3264, 0.1 μM LY294002, and 10 μM JSH-23 for 24 h. The expression levels of PI3K, AKT1, p65, their corresponding phosphorylated forms, and Cyclin D1 were assessed in cell lysates using western blotting analysis. **K-M** Relative intensities of all phosphorylated proteins were calculated after normalization to total protein. **N** The relative intensity of Cyclin D1 was calculated after normalization with HSP90 expression. Data are presented as mean ± SEM from three independent experiments. *p < 0.05, **p < 0.01, and ***p < 0.001, compared with levels in the untreated group or the group administered alone.

## Data Availability

Data will be made available on request.

## Abbreviations

TNBC: Triple-negative breast cancer
PI3K: Phosphatidylinositol 3-Kinase
AKT: Protein kinase B
NF-κB: Nuclear factor-kappa B
TCM: Traditional Chinese medicine
ER: Estrogen
PR: Progesterone
HER2: Human epidermal growth factor receptor-2
LDH: Lactate dehydrogenase
DEGs: Differential expression genes
GO: Gene ontology
KEGG: Kyoto encyclopedia of genes and genomes
BC: Breast cancer
mTORC1: Mammalian target of rapamycin complex 1
ALT: Alanine aminotransferase
AST: Aspartate aminotransferase
BUN: Blood urea nitrogen
CRE: Creatinine

## References

[1] Zongyuan Z, Jian W, Xiao M. The research progress of Pangolin. Pharmacy and Clinics of Chinese Materia Medica. 2014.

[2] Ywgzw HA. The research progress of application and preparation for endangered TCM pangolins. Mod Chin Med. 2015;17(03):280–4.

[3] Li M. Pharmacological and clinical studies on Pangolin. Res Tradit Chin Med. 2002;02:46–7.

[4] Wanjun X, Yi L, Shaowen Z, Honglin S, Xiaoyan L, Huayu Z. Analysis of medication regularity of Professor LIN Yi in treatment of granulomatous mastitis based on data mining. China Med Herald. 2019;16(03):120–3.

[5] Dongmei X. Pangolins in the treatment of cyclomastopathy. China Med Pharm. 2012;2(20):86–7.

[6] Ming-yue Z, Zhi-gang L. Analysis on the clinical profiling of current prominent TCM doctors on treatment of breast cancer. China J Tradit Chin Med Pharms. 2019;34(07):3162–6.

[7] Peng J. Study on the ecological geographical distribution, habitat selection and wild resources of Manis pentadactyla [Master]: Chongqing Normal University; 2021.

[8] Choo SW, Rayko M, Tan TK, Hari R, Komissarov A, Wee WY, et al. Pangolin genomes and the evolution of mammalian scales and immunity. Genome Res. 2016;26(10):1312–22. 27510566 10.1101/gr.203521.115 PMC5052048

[9] Xun L, Rui L, Cheng-Lei Z, Zhi-Yuan WU, Ye Z, Ru-Lan T, et al. Study on synergistic mechanism of pangolin in high temperature sand-fried processing. Chin Tradit Herb Drugs. 2019.

[10] So JY, Ohm J, Lipkowitz S, Yang L. Triple negative breast cancer (TNBC): non-genetic tumor heterogeneity and immune microenvironment: Emerging treatment options. Pharmacol Ther. 2022;237:108253. 35872332 10.1016/j.pharmthera.2022.108253 PMC9378710

[11] Alausa A, Victor UC, Fadahunsi OS, Owolabi N, Adeniji A, Olatinwo M, et al. Checkpoints and immunity in cancers: role of GNG12. Pharmacol Res. 2022;180:106242. 35513227 10.1016/j.phrs.2022.106242

[12] Ben-Abdallah M, Sturny-Leclère A, Avé P, Louise A, Moyrand F, Weih F, et al. Fungal-induced cell cycle impairment, chromosome instability and apoptosis via differential activation of NF-κB. PLoS Pathog. 2012;8(3):e1002555. 22396644 10.1371/journal.ppat.1002555 PMC3291658

[13] Wang J, Jia Z, Zhang Z, Wang Y, Liu X, Wang L, et al. Analysis of chemical constituents of Melastoma dodecandrum Lour. by UPLC-ESI-Q-Exactive Focus-MS/MS. Molecules. 2017;22(3):476. 28304342 10.3390/molecules22030476 PMC6155390

[14] Liu Y, He X, Di Z, Du X. Study on the active constituents and molecular mechanism of Zhishi Xiebai Guizhi decoction in the treatment of CHD based on UPLC-UESI-Q Exactive focus, gene expression profiling, network pharmacology, and experimental validation. ACS Omega. 2022;7(5):3925–39. 35155889 10.1021/acsomega.1c04491 PMC8829943

[15] Wang Y, Zhang Y, Xu P, Guo B, Li W. Metabolism distribution and effect of thiamethoxam after oral exposure in Mongolian racerunner (Eremias argus). J Agric Food Chem. 2018;66(28):7376–83. 29923398 10.1021/acs.jafc.8b02102

[16] Tu SH, Chiou YS, Kalyanam N, Ho CT, Chen LC, Pan MH. Garcinol sensitizes breast cancer cells to Taxol through the suppression of caspase-3/iPLA(2) and NF-κB/Twist1 signaling pathways in a mouse 4T1 breast tumor model. Food Funct. 2017;8(3):1067–79. 28145547 10.1039/C6FO01588C

[17] Yang W, Kang Y, Zhao Q, Bi L, Jiao L, Gu Y, et al. Herbal formula Yangyinjiedu induces lung cancer cell apoptosis via activation of early growth response 1. J Cell Mol Med. 2019;23(9):6193–202. 31237749 10.1111/jcmm.14501 PMC6714142

[18] Kim EJ, Choi MR, Park H, Kim M, Hong JE, Lee JY, et al. Dietary fat increases solid tumor growth and metastasis of 4T1 murine mammary carcinoma cells and mortality in obesity-resistant BALB/c mice. Breast Cancer Res BCR. 2011;13(4):R78. 21834963 10.1186/bcr2927 PMC3236342

[19] De La Rochere P, Guil-Luna S, Decaudin D, Azar G, Sidhu SS, Piaggio E. Humanized mice for the study of immuno-oncology. Trends Immunol. 2018;39(9):748–63. 30077656 10.1016/j.it.2018.07.001

[20] Wu C, Wang X, Shang H, Wei H. Construction of a humanized PBMC-PDX model to study the efficacy of a bacterial marker in lung cancer immunotherapy. Dis Markers. 2022;2022:1479246. 36072895 10.1155/2022/1479246 PMC9441396

[21] Wu Q, Chen DQ, Sun L, Huan XJ, Bao XB, Tian CQ, et al. Novel bivalent BET inhibitor N2817 exhibits potent anticancer activity and inhibits TAF1. Biochem Pharmacol. 2021;185:114435. 33539817 10.1016/j.bcp.2021.114435

[22] Wang J, Chen GL, Cao S, Zhao MC, Liu YQ, Chen XX, et al. Adipogenic niches for melanoma cell colonization and growth in bone marrow. Lab Investig J Tech Methods Pathol. 2017;97(6):737–45. 10.1038/labinvest.2017.14 28218738

[23] Dutta S, Kharkar PS, Sahu NU, Khanna A. Molecular docking prediction and in vitro studies elucidate anti-cancer activity of phytoestrogens. Life Sci. 2017;185:73–84. 28720470 10.1016/j.lfs.2017.07.015

[24] Honmore VS, Kandhare AD, Kadam PP, Khedkar VM, Natu AD, Rojatkar SR, et al. Diarylheptanoid, a constituent isolated from methanol extract of Alpinia officinarum attenuates TNF-α level in Freund’s complete adjuvant-induced arthritis in rats. J Ethnopharmacol. 2019;229:233–45. 30336303 10.1016/j.jep.2018.10.019

[25] Kumar P, Nagarajan A, Uchil PD. Analysis of cell viability by the lactate dehydrogenase assay. Cold Spring Harb Protoc. 2018;2018(6):465–8. 10.1101/pdb.prot095497 29858337

[26] Wang Y, Kuramitsu Y, Baron B, Kitagawa T, Tokuda K, Akada J, et al. PI3K inhibitor LY294002, as opposed to wortmannin, enhances AKT phosphorylation in gemcitabine-resistant pancreatic cancer cells. Int J Oncol. 2017;50(2):606–12. 28000865 10.3892/ijo.2016.3804

[27] Abdul-Ghani R, Serra V, Györffy B, Jürchott K, Solf A, Dietel M, et al. The PI3K inhibitor LY294002 blocks drug export from resistant colon carcinoma cells overexpressing MRP1. Oncogene. 2006;25(12):1743–52. 16288223 10.1038/sj.onc.1209201

[28] Guo L, Li C, Guo J, Qiu J, Hua K. CKAP2 promotes cervical cancer progression by modulating the tumor microenvironment via NF-κB signaling. Am J Cancer Res. 2023;13(6):2376–91. 37424820 PMC10326565

[29] Ahmed KM, Zhang H, Park CC. NF-κB regulates radioresistance mediated by β1-integrin in three-dimensional culture of breast cancer cells. Can Res. 2013;73(12):3737–48. 10.1158/0008-5472.CAN-12-3537 PMC3698967 23576567

[30] Chai XP, Sun GL, Fang YF, Hu LH, Liu X, Zhang XW. Tumor-targeting efficacy of a BF211 prodrug through hydrolysis by fibroblast activation protein-α. Acta Pharmacol Sin. 2018;39(3):415–24. 29119969 10.1038/aps.2017.121 PMC5843830

[31] Khan M, Maryam A, Qazi JI, Ma T. Targeting apoptosis and multiple signaling pathways with icariside ii in cancer cells. Int J Biol Sci. 2015;11(9):1100–12. 26221076 10.7150/ijbs.11595 PMC4515820

[32] Bai C, Zhao J, Su J, Chen J, Cui X, Sun M, et al. Curcumin induces mitochondrial apoptosis in human hepatoma cells through BCLAF1-mediated modulation of PI3K/AKT/GSK-3β signaling. Life Sci. 2022;306:120804. 35882275 10.1016/j.lfs.2022.120804

[33] Weng W, Goel A. Curcumin and colorectal cancer: an update and current perspective on this natural medicine. Semin Cancer Biol. 2022;80:73–86. 32088363 10.1016/j.semcancer.2020.02.011 PMC7438305

[34] He L, Pan Y, Yu J, Wang B, Dai G, Ying X. Decursin alleviates the aggravation of osteoarthritis via inhibiting PI3K-Akt and NF-kB signal pathway. Int Immunopharmacol. 2021;97:107657. 33878544 10.1016/j.intimp.2021.107657

[35] Joyce D, Albanese C, Steer J, Fu M, Bouzahzah B, Pestell RG. NF-kappaB and cell-cycle regulation: the cyclin connection. Cytokine Growth Factor Rev. 2001;12(1):73–90. 11312120 10.1016/S1359-6101(00)00018-6

[36] Xie L, Chen T, Qi X, Li H, Xie J, Wang L, et al. Exopolysaccharides from Genistein-stimulated Monascus purpureus ameliorate cyclophosphamide-induced intestinal injury via PI3K/AKT-MAPKs/NF-κB pathways and regulation of gut microbiota. J Agric Food Chem. 2023;71(35):12986–3002. 37611142 10.1021/acs.jafc.3c03186

