# Supplementary figures and images for: Dipeptide PA3264 derived from rare and endangered Squama Manis is a novel bioactive peptide for the treatment of triple-negative breast cancer
Source: Chin Med. 2024 Aug 21;19:112. doi: 10.1186/s13020-024-00979-x (PMC11340106; doi:10.1186/s13020-024-00979-x)

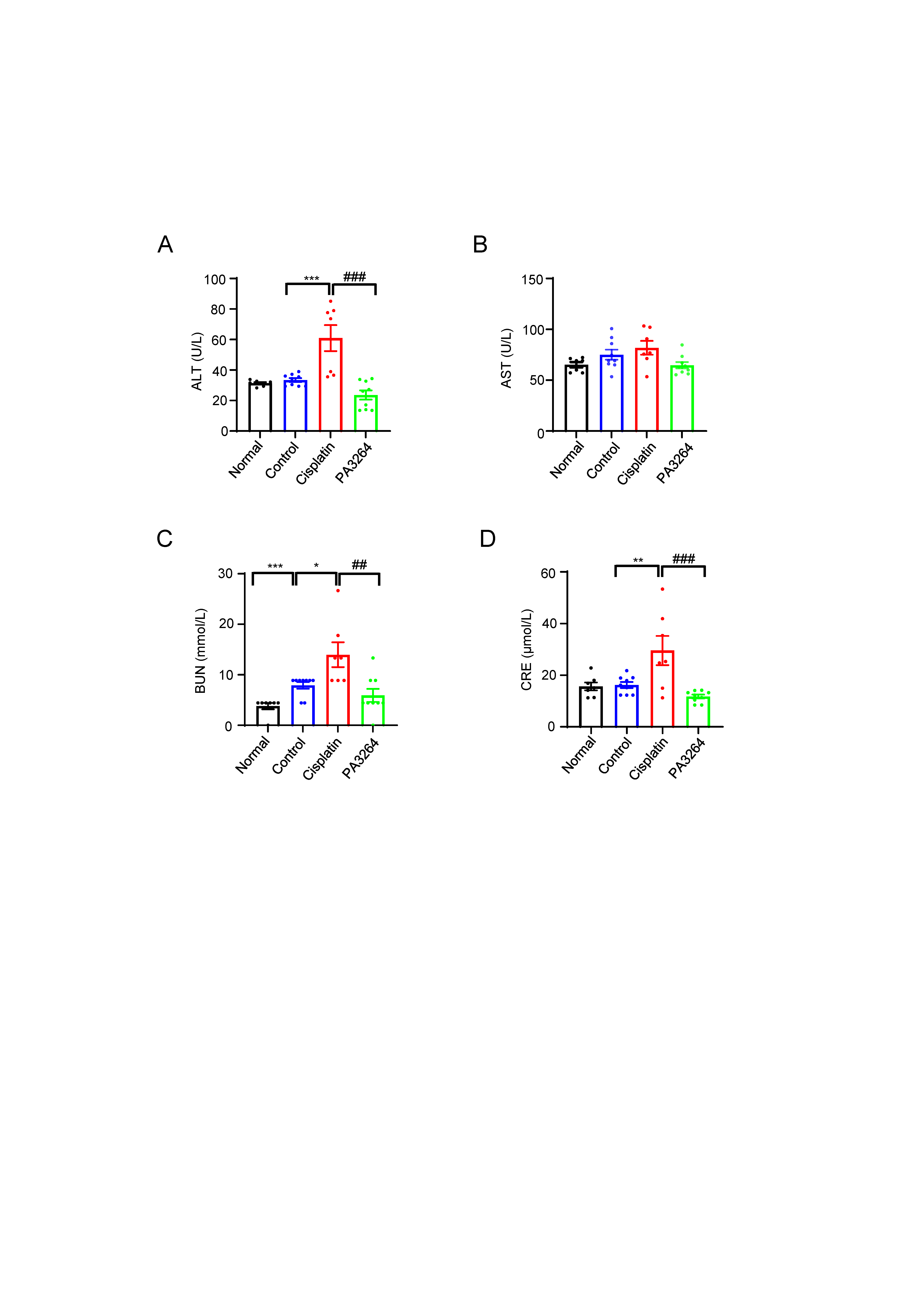

Supplement: Supplementary file 1 — Supplementary material 1: Fig. 1 Effects on liver and renal function indicators in various groups of mice. A Alanine aminotransferase (ALT), B aspartate aminotransferase (AST), C blood urea nitrogen (BUN), and D creatinine (CRE) levels in the serum were measured. The figure shows the mean ± SEM of the experimental data for each group. Compared with the normal and control groups, *p < 0.05, **p < 0.01, ***p < 0.001. Compared with the cisplatin group, #p < 0.05, ##p < 0.01, ###p < 0.001, n = 7-9. [file 13020_2024_979_MOESM1_ESM.tiff]

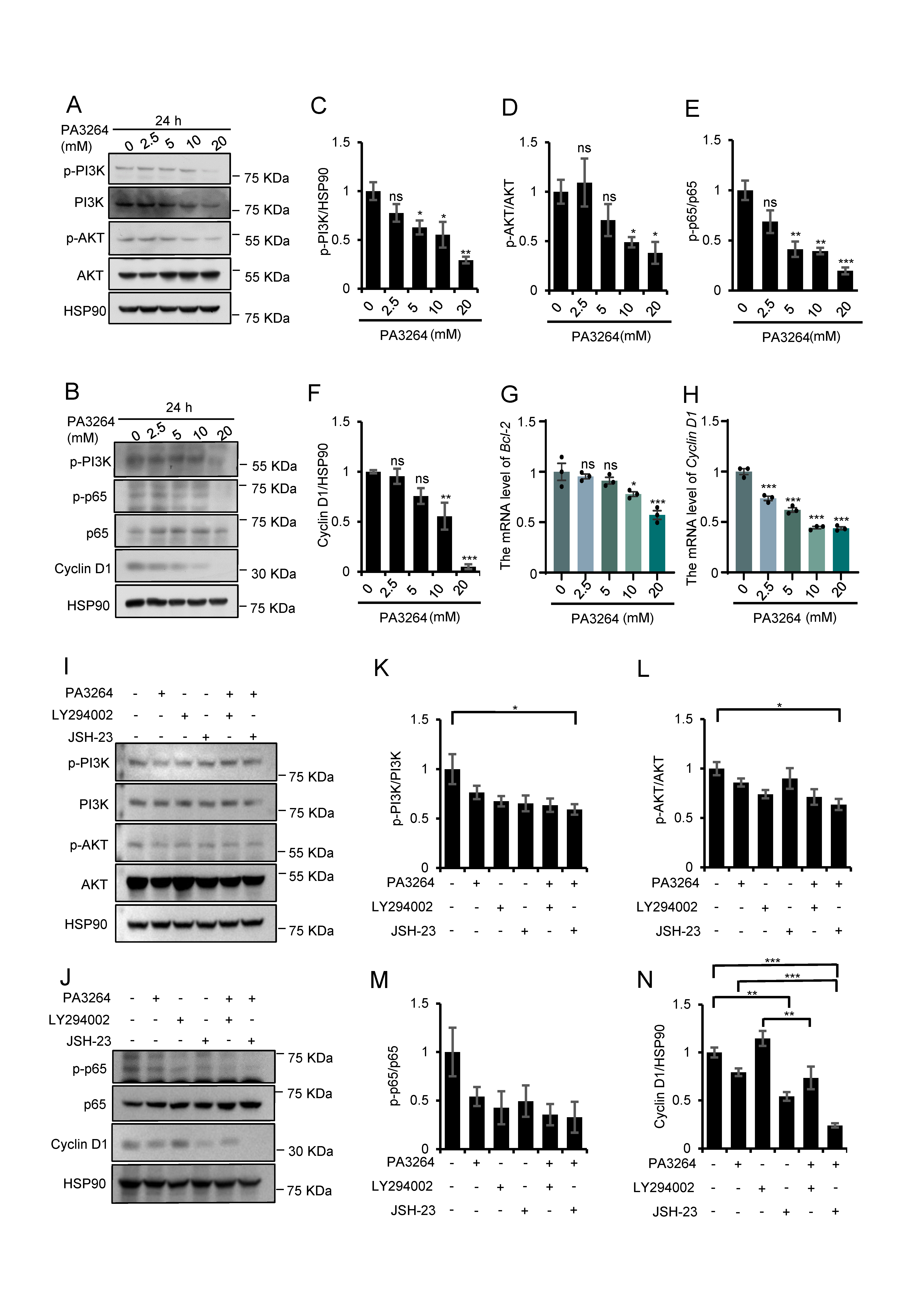

Supplement: Supplementary file 2 — Supplementary material 2: Fig. 2 Effect of PA3264 with pathway inhibitors on PI3K/AKT/NF-κB signaling in MDA-MB-231 cells. A-B MDA-MB-231 cells were treated with the indicated concentrations of PA3264 for 24 h, and the expression levels of PI3K, AKT1, p65, Cyclin D1, and all phosphorylated forms in total cell lysates were evaluated by western blot analysis. C-E The relative intensities of all phosphorylated proteins were calculated after normalization to the total proteins. F Relative intensities of Cyclin D1 were calculated after normalization to HSP90 expression. G-H Analysis of Cyclin D1 and Bcl-2 expression in MDA-MB-231 cells treated with PA3264 at different concentrationsusing qRT-PCR. I-J MDA-MB-231 cells were treated with 20 mM PA3264, 0.1 μM LY294002, and 10 μM JSH-23 for 24 h. The expression levels of PI3K, AKT1, p65, their corresponding phosphorylated forms, and Cyclin D1 were assessed in cell lysates using western blotting analysis. K-M Relative intensities of all phosphorylated proteins were calculated after normalization to total protein. N The relative intensity of Cyclin D1 was calculated after normalization with HSP90 expression. Data are presented as mean ± SEM from three independent experiments. *p < 0.05, **p < 0.01, and ***p < 0.001, compared with levels in the untreated group or the group administered alone. [file 13020_2024_979_MOESM2_ESM.tiff]
